# Annotations

**Published:** 1895-09-28

**Authors:** 


### ANNOTATIONS.

### London's Mental Wreckage.

The waste products of a crowded life form one of the embarrassments of civilisation. Every centre of population becomes surrounded by a suburban ring of refuse-heaps; kitchen gardens in which at least waste is turned to good use, reformatories where some attempt at any rate is made to restore to good citizenship those who by city life have been led astray, workhouses, prisons, refuges, and—perhaps saddest of all—asylums in which so many have to live out their lives, drifting year by year towards dementia. The vastness of the population of London, the wealth, the trade, the world-wide importance of this great city, are subjects of unending interest, and to some are matters of continual satisfaction. But there is a seamy side, the tares spring up even more quickly than the wheat, and the timorous may well fear lest the good seed may not be choked by the weeds which thrive so vigorously in our midst. It is no small matter that the pauper lunatics of London should increase at the rate of five hundred to six hundred a year. No sooner has the pressure on metropolitan asylums been temporarily relieved by the opening of a splendid hospital for the insane at Claybury than we find the lunatic population crowding on the heels of those who provide for it, so that, according to the report recently issued by the London County Council, there is at the present moment a deficiency in the accommodation at their disposal to the extent of 1,317 beds, the total number of lunatics chargeable to the various parishes and unions in the metropolis on January 1st, 1895, being 18,541. To overtake the deficiency of accommodation which now exists a new asylum is at once to be erected at Bexley to receive 2,000 patients, at an expense, exclusive of the cost of site and equipment, of £350,000. This is a goodly sum, amounting to £175 per bed, or at least £200 per bed when land and furnishing are included. For a hospital this is not excessive, although we may doubt whether all the patients who go to these asylums either require or get what may properly be called hospital treatment. But we do not grumble at the price if these poor wastrels do but get reasonable care, and if a scientific effort is made to cure them of their ailments, although we may question whether such vast establishments are the best means to such an end. What seems, however, so hopeless and so sad is that 1,300 of these 2,000 beds are already wanted, and that, at the present rate of increase in the number of metropolitan pauper lunatics, long before the new asylum is open every bed will be engaged, and we shal be again face to face with this perennial problem of providing for the mental wreckage which hangs at the fringe of our boasted progress.

### Fresh Air for Old Folks.

We have everywhere societies for sending children to the country or the seaside for longer or shorter periods, and everyone who has seen their working admits their value. Besides the gain in health and strength, there is an increase in intelligence, a realisation of a life outside the gutter and the slum, which is full of good, But would it not be worth our while to try a "fresh-air fortnight" for middle-aged and old people? To all, but especially to those who were brought up in the country, though they found their destiny in the town, there must come a longing for pure air and a blue sky which they cannot gratify unaided. When one sees how many avail themselves of the dubious felicity of a Bank holiday excursion, one cannot doubt that a few days in the country would be regarded as a boon. Of course it is not so easy for fathers and mothers to get away from home as it is for the children. But most women have some relative who would take care of the family for a few days, and there are employers who will give a man a week's holiday without turning him out of his job, for the average employer is not so black as he is painted. And even if those in active life cannot always take a holiday there are the old folks who sit by the fireside and "babble o' green fields" which they remember in their youth. The grandmother may help to look after the children, the grandfather do an odd bit of work now and then, but they could be spared for a few days if a home could be found for them and there was not much expense connected with their holiday. And how the little change would cheer the old people, who have not much pleasure in the decline of their laborious life. It must be admitted that a caravan of old people entails more anxiety on the part of those who take charge of them than the same number of children, but "old" does not imply merely the wrinkled beldame and slippered pantaloon, but those who have discharged their duty by bringing up children to work in the world. The science of charity, which looks to taking those whom it helps out of the range of requiring aid, may not have much to say to them; but in the gentle act of charity, which simply tries to help the poor, they have their place.

### The Friends of Man.

To be the friend of man is not all gain. Whether by a process of evolution or degeneration, many animals have become truly domesticated. They have come to depend on man for food, for shelter, for protection On the other hand, the more closely they have lived with him, and especially the more they have adopted the shelter of his roof, the more they have become exposed to his great enemy—tuberculosis. Professor Delépine, writing on this subject, has shown that—taking very large numbers as the basis of his estimate—at least 16 per cent. of cattle are afflicted with this disease; and that, whereas in some districts it may be comparatively rare, there are parts in which a non-tuberculous cow is the exception. Pigs also are affected in the same manner, although not to the same extent about one in every 36 being attacked by the disease. Cats and dogs also are subject to tuberculosis, and it is to be feared from their exceeding friendliness may be a source of danger to children with whom they play. Although the form of tuberculosis with which poultry are affected differs in some particulars from that of man, it is a very common disease, and commits great ravages in poultry yards. So far we have dealt with friends of man, which have so far adopted his habits as to dwell under the shelter of a roof. In the case, however, even of sheep which are rarely under shelter, and in many respects live in more natural conditions, tuberculosis has been found; and, in fact, the only real friend of man which has almost entirely escaped the ravages of tuberculosis is the goat. But any animal which conforms with man's habit of dwelling under artificial shelter is apt to contract tuberculosis, and so it is that whether they be monkeys, camels, giraffes, antelopes, llamas, lions, tigers, foxes, tapirs, zebras, &c., they all, according to Professor Delépine, are liable to tuberculosis when they are kept in menageries. Clearly the friendship of man is not an unmitigated blessing.

